# Mothers do it differently: reproductive experience alters fear extinction in female rats and women

**DOI:** 10.1038/tp.2016.193

**Published:** 2016-10-25

**Authors:** J S Milligan-Saville, B M Graham

**Affiliations:** 1 School of Psychology, The University of New South Wales Australia, Sydney, NSW, Australia

## Abstract

Fear extinction is the laboratory basis of exposure therapy for anxiety disorders. Recent findings have revealed that estradiol is necessary to the consolidation of extinction memories in females. These findings are based on studies conducted using virgin rats and young women whose reproductive history is unknown. We hypothesized that motherhood, which results in extensive endocrinological, neurobiological and behavioral changes, may lead to alterations in fear extinction in females. We used a cross-species translational approach to investigate the impact of reproductive experience on fear extinction and fear relapse in female rats (*n*=116) and women (*n*=64). Although freezing during extinction recall was associated with estrous cycle phase during extinction training in virgin rats, this association was mitigated in age-matched reproductively experienced rats, even when fear extinction occurred 3 months after pups had been weaned, and even though reproductively experienced rats exhibited attenuated serum estradiol levels. In addition, although serum estradiol levels predicted extinction recall in human women with no prior reproductive experience, no such association was found in women with children. Finally, although virgin rats displayed both renewal and reinstatement after fear extinction, these common relapse phenomena were absent in rats with reproductive experience. Together, these findings suggest that reproductive experience alters the endocrine and behavioral features of fear extinction in females long after the hormonal surges of pregnancy and lactation have diminished. These results highlight the need to incorporate both hormonal and reproductive status as important factors in current models of fear extinction in females.

## Introduction

Anxiety disorders are characterized by deficits in fear inhibition, which can be studied in rats and humans using fear extinction.^1^ In this procedure, the subjects are repeatedly presented a feared conditioned stimulus (CS; for example, a noise that was previously paired with shock) in the absence of the aversive unconditioned stimulus (US; that is, the shock). Extinction recall is assessed by re-presenting the extinguished CS, with low levels of fear indicating robust recall. However, fear can return if the CS is presented in a different context to extinction (renewal) or following an unsignalled US (reinstatement). Exposure therapy, the gold-standard psychological treatment for anxiety disorders, was based on extinction.^2^ Identifying the factors associated with individual differences in extinction and fear return may lead to means of enhancing exposure therapy and reducing post-treatment relapse.

The current cross-species model of extinction is limited by the fact that it does not take into account gender or sex hormone fluctuations.^3, 4^ Recent findings have revealed that naturally cycling female rats^5, 6, 7, 8^ and women^9, 10^ extinguished during the high estradiol phase of the estrous/menstrual cycle exhibited strong extinction recall. In contrast, subjects extinguished during the low estradiol phases demonstrated return of fear during extinction recall. Administration of estradiol^5, 7, 10, 11, 12^ or estrogen receptor (ER) agonists^10, 11, 12^ enhanced extinction recall, whereas hormonal contraceptives (which reduce serum estradiol levels)^12^ or ER antagonists^7^ impaired extinction recall. High levels of endogenous estradiol enhanced amygdala, medial prefrontal cortex and hippocampus^10^ activity; components of the extinction neural circuitry.^13^ Collectively, these results suggest that high levels of estradiol facilitate extinction memory consolidation by modulating ER activity within this neural circuitry.

The role of estradiol in extinction has been examined using virgin female rats and young women (mean age ≈23) whose reproductive status is unknown; however, the majority of women spent at least half of their lives as mothers.^14^ In rats and humans, reproductive experience (from pregnancy through to the rearing of offspring) results in extensive endocrinological, neurobiological and behavioral changes,^15, 16, 17, 18^ many of which persist long after nursing has ceased. Compared with nulliparous (that is, no reproductive experience) rats, parous (that is, at least one prior reproductive experience) rats exhibit attenuated stress-induced amygdala activity, attenuated behavioral responsiveness to threat and increased hippocampus-dependent memory and plasticity, even up to 18 months after pups had been weaned.^19^ Similarly, even 9 months post weaning, reproductive experience abolished the detrimental impact of stress on eyeblink conditioning observed in nulliparous rats.^20^ Though there are inconsistencies in the literature, long-term modifications of learning and memory have also been demonstrated in human mothers.^21, 22^

Given that motherhood substantially changes the function of neural structures involved in extinction, behavioral responses to threat, and learning/memory capacity, we hypothesized that reproductive experience may alter extinction in females. We compared extinction recall in female rats and women extinguished during high and low estradiol phases of their cycle (experiments 1 and 4), and tested whether reproductive experience and estrous phase influence renewal and reinstatement (experiments 2 and 3). We report substantive changes in both the hormonal and behavioral features of fear extinction associated with reproductive experience.

## Materials and methods

### Animal subjects

**Figure 1:**
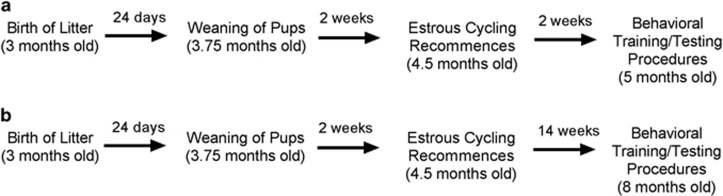
Experimental timeline for primiparous rats in experiment 1 (**a**) and experiments 2 and 3 (**b**). Note that virgin rats were age-matched to primiparous rats in each experiment.

Virgin, female, Sprague Dawley rats (8–10 weeks old), obtained from the Animal Resource Centre, Perth, WA, Australia were housed in groups of 7–10 at the UNSW School of Psychology, Australia. The rats were randomly assigned to remain as virgins or to be mated. During breeding, primiparous rats were housed in groups of four to five with one sexually experienced male rat. After 2.5 weeks, pregnant rats were individually housed until the birth of their litter, and remained housed with their pups until weaning at postnatal day 24 (see Supplementary Information for details). Nulliparous and primiparous rats were age-matched in each of the experiments, and underwent fear conditioning at 5 months of age (experiment 1) or 8 months of age (experiments 2 and 3). See Figure 1 for a timeline of experimental procedures (from birth of litter to behavioral training and testing) for primiparous rats. Sample sizes were determined on the basis of medium–large effect sizes in previous investigations of estrous cycle effects on fear extinction.^5^ All the procedures were approved by the UNSW Animal Care and Ethics Committee.

### Estrous cycle phase determination

Vaginal smears were conducted to determine estrous cycle phase as previously described.^5^ Only rats with a regular 4-day estrous cycle were included. Primiparous rats were not fear conditioned until at least 2 weeks after weaning when estrous cycling had recommenced.^23^

Rats underwent extinction training during proestrus (high estradiol) or metestrus (low estradiol), as these phases have a differential effect on extinction recall.^7^ The three experimental phases were run 24 h apart, and so rats were fear conditioned during diestrus (proestrus group) or estrus (metestrus group), and tested for extinction recall during estrus (proestrus group) or diestrus (metestrus group). Previous research has shown that the difference in extinction recall between proestrus and metestrus groups occurs even when estrous phase was held constant during fear conditioning and extinction recall.^5^

### Animal apparatus and procedures

The apparatuses were identical to those previously described,^5^ and comprised two sets of chambers that served as distinct contexts (A and B). The CS was a white noise (4 dB above background) and the US was a scrambled foot-shock (1s, 0.4 mA). A brief (1–2 min) adaptation period to the context preceded CS presentations during all experimental phases.

#### Fear conditioning

The rats underwent fear conditioning in Context A, consisting of two 10 s CSs co-terminating with the US (intertrial interval: 85–135 s; mean intertrial interval: 110 s).

#### Extinction training

Twenty-four hours after fear conditioning, the rats underwent extinction training in Context B, consisting of 30 non-reinforced 10 s CSs (intertrial interval: 10 s).

#### Recall

Twenty-four hours after extinction training, the rats were tested for extinction recall, consisting of a single non-reinforced 2 min CS. In experiment 1, recall was performed in the extinction training context B. In experiment 2, the rats were tested in Context B and then again 1 h later in the fear conditioning context A to evaluate renewal. In experiment 3, the rats were tested in Context B twice; 24 h after extinction training, and then 24 h after an unsignalled US presentation, to assess reinstatement.

#### Reinstatement

In experiment 3 only, 24 h following the first extinction recall test, the rats were returned to Context B and exposed to one unsignalled US presentation (0.5 s, 0.3 mA).

### Behavioral data analysis

The percentage of time spent freezing, defined as the absence of all movement excluding that required for respiration, was the measure of conditioned fear.^24^ The data for extinction training are presented as six blocks of trials, each representing an average of five trials.

### Serological estradiol analysis (rats)

Three days after the completion of behavioral procedures, the rats from experiment 2 were killed and trunk blood was collected. The serum was analyzed for estradiol concentration using a commercially available enzyme-linked immunosorbent assay kit (ab108667, Abcam, Melbourne, VIC, Australia) according to the manufacturer's instructions.

### Human participants

Premenopausal, naturally cycling women (18–48 years old) without endocrinological conditions, with or without children, were recruited through advertisement from the local community. Written informed consent was obtained from all the participants in accordance with the UNSW Human Research Ethics Committee.

### Serological estradiol analysis (humans)

Women were invited to participate in the experiment across all phases of the menstrual cycle to achieve wide variance in estradiol levels. A blood sample was drawn from each participant approximately 15 min after extinction training for the assessment of serum estradiol (see Supplementary Information for details).

### Human conditioning and extinction procedures

A 2-day differential fear conditioning and extinction procedure was used in a single context (see Supplementary Information for details). This 2-day procedure is similar to that used in previous research on the influence of estradiol on fear extinction in women.^9, 10, 12^ The CSs were two photographs of male faces with neutral expressions, and were presented in a pseudo-random order throughout the experiment. The US was a mild electric shock (0.5 s), the level of which was selected by each participant to be highly annoying but not painful, delivered to the dominant hand. On Day 1, the participants underwent habituation, consisting of two non-reinforced presentations of each CS. Conditioning commenced immediately after habituation, consisting of eight presentations of each CS. One face (CS+) was reinforced by shock on 62.5% of trials, and the other face was never reinforced (CS−). The CS+ was counterbalanced within the reproductive experience groups. Extinction training followed immediately after conditioning, consisting of seven non-reinforced presentations of each CS. On Day 2 (approximately 24 h later), the participants were tested for extinction recall, and this was identical to extinction training.

### Psychophysiological data analysis

Skin conductance responses (SCRs) were used as the measure of conditioned fear. Conditioning strength was indexed as the average differential SCRs across conditioning (average SCRs to the CS+ minus average SCRs to the CS−). Extinction acquisition and extinction recall were indexed as the percentage of fear remaining at the end of extinction training or during recall, as a function of conditioning strength. This was achieved by dividing the average SCRs to the CS+ during the last two extinction trials, or during the first two extinction recall trials, by the maximum SCR to the CS+ during conditioning, and multiplying the result by 100 (see Supplementary Information for details).

### Statistical analysis

The data were analyzed using SPSS, Version 22 (IBM, Armonk, NY, USA). For experiments 1–3, two-way analyses of variances assessed group differences in baseline freezing during fear conditioning, extinction training and extinction recall (presented in Supplementary Table S1), as well as CS-elicited freezing during extinction recall. Two-way analyses of variances with repeated measures assessed group differences in CS-elicited freezing during fear conditioning and extinction training. *Post hoc* independent samples *t*-tests and Fisher's Least Significant Difference tests were used when appropriate.

For experiment 4, one-way analyses of variances assessed group differences in age, shock level and unconditioned responses, and SCRs during experimental phases. Correlational analyses assessed the relationship between estradiol levels and SCRs during all experimental phases. A multiple regression analysis assessed the interaction between estradiol levels and reproductive experience, controlling for confounding variables. Three statistical outliers (defined as >4 s.d. away from the mean) were removed from the analysis of experiments 1 and 4, and one statistical outlier was removed from the serum estradiol analysis of experiment 2 (see Supplementary Information for details).

## Results

### Experiment 1: reproductive experience attenuates the association between estrous cycle phase and extinction recall in female rats

**Figure 2:**
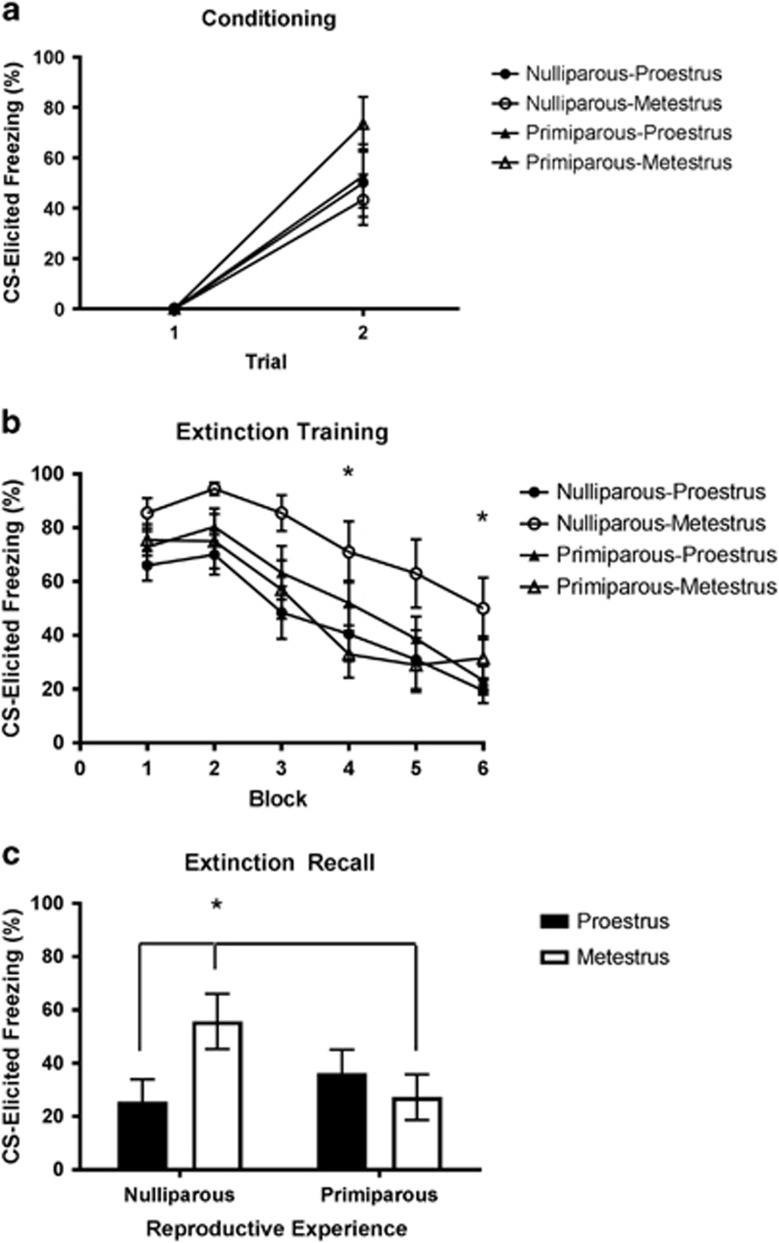
(**a**) Mean (±s.e.m.) conditioned stimulus (CS)-elicited freezing for groups nulliparous-proestrus (*n*=10), nulliparous-metestrus (*n*=10), primiparous-proestrus (*n*=12) and primiparous-metestrus (*n*=10) during fear conditioning in experiment 1. (**b**) Mean (±s.e.m.) CS-elicited freezing during extinction training in experiment 1 (each block represents an average of five trials). *Nulliparous-metestrus > nulliparous-proestrus and primiparous-metestrus (*P*<0.05). (**c**) Mean (±s.e.m.) CS-elicited freezing during extinction recall in experiment 1. *Nulliparous-proestrus < nulliparous-metestrus and primiparous-metestrus (*P*<0.05).

Experiment 1 investigated the influence of reproductive experience on the effect of estrous cycle on extinction recall in age-matched nulliparous and primiparous rats, 2 weeks after pups had been weaned. Freezing increased across fear conditioning (F(1,38)=84.05, *P*<0.001; Figure 2a). There were no significant main effects of reproductive experience or estrous cycle, and no significant reproductive experience × estrous cycle interaction (largest F(1,38)=1.88, *P*=0.18). Freezing decreased across extinction training (F(5,190)=39.3, *P*<0.001; Figure 2b). There were no significant main effects of reproductive experience or estrous cycle (largest F(1,38)=3.47, *P*=0.07). There was a significant reproductive experience × estrous cycle interaction (F(1,38)=6.93, *P*=0.012), owing to nulliparous-metestrus rats displaying higher freezing than primiparous-metestrus and nulliparous-proestrus rats on the fourth and final blocks of extinction training, respectively (largest *P*=0.047). There was no significant group difference during early extinction, suggesting comparable memory for fear conditioning (F(3,38)=1.83, *P*=0.158). During extinction recall (Figure 2c), there were no significant main effects of reproductive experience or estrous cycle (largest F(1,38)=1.35, *P*=0.25); however, there was a significant reproductive experience × estrous cycle interaction (F(1,38)=4.6, *P*=0.038). This was owing to nulliparous-metestrus rats freezing significantly more than nulliparous-proestrus and primiparous-metestrus rats (largest *P*=0.037); all other groups did not differ significantly from one another (smallest *P*=0.132). These data indicate that reproductive experience mitigates the association between estrous cycle and extinction recall, without altering fear conditioning or within-session extinction.

### Experiment 2: renewal of conditioned fear is abolished by reproductive experience in female rats

Experiment 2 sought to replicate and extend the findings of experiment 1 by assessing the impact of reproductive experience on extinction recall and renewal in age-matched nulliparous and primiparous rats, 3 months after pups had been weaned. This was to explore whether the mitigated association between estrous cycle and fear extinction persists over a longer period of time post weaning, and whether reproductive experience alters the behavioral features of fear extinction. Moreover, serum estradiol levels were assessed 3 days after test for extinction recall to determine whether reproductive experience is associated with an altered endocrine profile. This time point was chosen to match the estrous phase during which the rats received extinction training; thus, it provides an indication of the relative levels of serum estradiol that would have been present in the different groups at the time of extinction training.

**Figure 3:**
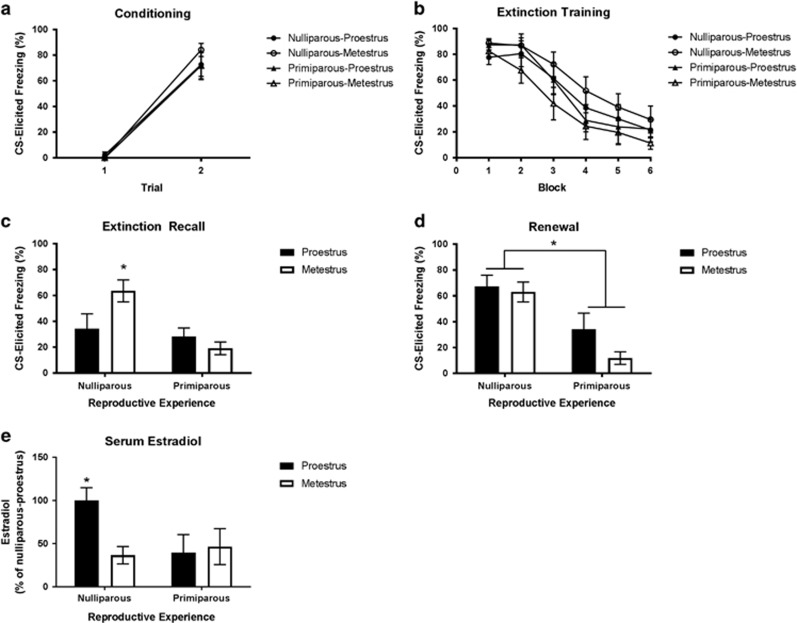
(**a**) Mean (±s.e.m.) conditioned stimulus (CS)-elicited freezing for groups nulliparous-proestrus (*n*=11), nulliparous-metestrus (*n*=11), primiparous-proestrus (*n*=9) and primiparous-metestrus (*n*=8) during fear conditioning in experiment 2. (**b**) Mean (±s.e.m.) CS-elicited freezing during extinction training in experiment 2 (each block represents an average of five trials). (**c**) Mean (±s.e.m.) CS-elicited freezing during extinction recall in experiment 2. *Nulliparous-metestrus > all other groups (*P*<0.05) in extinction training context. (**d**) Mean (±s.e.m.) CS-elicited freezing during test for renewal of fear in conditioning context in experiment 2. *Primiparous groups < nulliparous groups (*P*<0.05). (**e**) Mean (±s.e.m.) serum estradiol levels assessed 3 days after test for extinction recall and renewal, when the rats were in the same estrous cycle phase as they were in during extinction training. Estradiol levels are presented as a percentage of those obtained from the rats in the nulliparous-proestrus groups. *Nulliparous-proestrus > all other groups.

Freezing increased across fear conditioning (F(1,35)=284.91, *P*<0.001; Figure 3a). There were no significant main effects of reproductive experience or estrous cycle, and no significant reproductive experience × estrous cycle interaction (largest F(1,35)=0.63, *P*=0.44). Freezing decreased across extinction training (F(5,175)=61.88, *P*<0.001; Figure 3b). There were no main effects of reproductive experience or estrous cycle, and no reproductive experience × estrous cycle interaction (largest F(1,35)=1.9, *P*=0.176). During extinction recall (Figure 3c), there was no main effect of estrous cycle, but there was a significant effect of reproductive experience (F(1,35)=7.69, *P*=0.009), and a significant reproductive experience × estrous cycle interaction (F(1,35)=4.41, *P*=0.043). This was due to nulliparous-metestrus rats freezing more than all the other groups (largest *P*=0.021); all the other groups did not differ significantly from one another (smallest *P*=0.253). During renewal (Figure 3d), there was a main effect of reproductive experience (F(1,35)=21.38, *P*<0.001), but no main effect of estrous cycle and no reproductive experience × estrous cycle interaction (largest F(1,35)=2.16, *P*=0.15), due to primiparous rats freezing less than nulliparous rats. To compare renewal effects across the groups, a series of paired-sample *t*-tests were conducted, comparing changes in freezing from extinction recall to renewal within each group. Although nulliparous-proestrus rats exhibited renewal (*t*(10)=−4.36, *P*=0.001), primiparous rats exhibited no increase in freezing from the extinction recall context to the conditioning context (largest *t*=1.1). The nulliparous-metestrus rats continued to exhibit high freezing, unchanged from that during extinction recall (*t*(10)=0.056, *P*=0.96; note that ‘renewal' cannot be properly assessed in nulliparous-metestrus rats as they did not exhibit sufficient extinction recall in Context B).

Serum estradiol levels differed across groups (Figure 3e); although there were no main effects of reproductive experience or estrous phase (smallest *P*=0.102), there was a significant reproductive experience × estrous cycle interaction (F(1,34)=4.36, *P*<0.044). This was owing to nulliparous-proestrus rats exhibiting significantly greater estradiol levels than all other groups (largest *P*=0.032); all other groups did not differ significantly from one another (smallest *P*=0.68).

### Experiment 3: reinstatement of conditioned fear is abolished by reproductive experience in female rats

**Figure 4:**
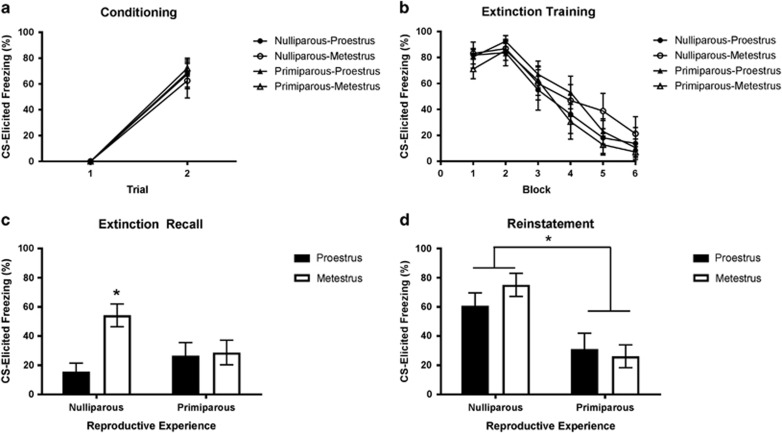
(**a**) Mean (±s.e.m.) conditioned stimulus (CS)-elicited freezing for groups nulliparous-proestrus (*n*=8), nulliparous-metestrus (*n*=8), primiparous-proestrus (*n*=10) and primiparous-metestrus (*n*=9) during fear conditioning in experiment 3. (**b**) Mean (±s.e.m.) CS-elicited freezing during extinction training in experiment 3 (each block represents an average of five trials). (**c**) Mean (±s.e.m.) CS-elicited freezing during extinction recall in experiment 3. *Nulliparous-metestrus > all other groups (*P*<0.05). (**d**) Mean (±s.e.m.) CS-elicited freezing during test for reinstatement of fear in experiment 3. *Primiparous groups < nulliparous groups (*P*<0.05).

Experiment 3 sought to replicate and extend the findings of the previous experiments by assessing the impact of reproductive experience on extinction recall and reinstatement in age-matched nulliparous and primiparous rats, three months after pups had been weaned. This was to investigate whether parous females show a generalized resistance to relapse following fear extinction. Freezing increased across fear conditioning (F(1,31)=162.89, *P*<0.001; Figure 4a). There were no significant main effects of reproductive experience or estrous cycle, and no significant reproductive experience × estrous cycle interaction (largest F(1,31)=0.27, *P*=0.61). Freezing decreased across extinction training (F(5,155)=63.65, *P*<0.001; Figure 4b). There were no main effects of reproductive experience or estrous cycle, and no reproductive experience × estrous cycle interaction during extinction training (largest F(1,31)=1.3, *P*=0.26). During extinction recall (Figure 4c), there was no main effect of reproductive experience, but there was a significant effect of estrous cycle (F(1,31)=6.34, *P*=0.017), and a significant reproductive experience × estrous cycle interaction (F(1,31)=5.06, *P*=0.032). This was owing to nulliparous-metestrus rats freezing less than all the other groups (largest *P*=0.036); all the other groups did not differ significantly from one another (smallest *P*=0.27). During the reinstatement test (Figure 4d), there was a main effect of reproductive experience (F(1,31)=18.38, *P*<0.001), but no main effect of estrous cycle and no reproductive experience × estrous cycle interaction (largest F(1,31)=1.1, *P*=0.3), owing to primiparous rats freezing less than nulliparous rats. To compare reinstatement effects across the groups, a series of paired-sample *t*-tests were conducted, comparing changes in CS-elicited freezing from extinction recall to reinstatement within each group. Although nulliparous-proestrus rats exhibited reinstatement (*t*(7)=−6.09, *P*<0.001), primiparous rats exhibited no increase in freezing from extinction recall to reinstatement test (all *t*<1). Nulliparous-metestrus rats continued to exhibit high levels of freezing, albeit significantly higher than that during extinction recall (*t*(7)=−2.48, *P*=0.042; note that ‘reinstatement' cannot be properly assessed in nulliparous-metestrus rats as they did not exhibit sufficient extinction recall at the outset).

### Experiment 4: reproductive experience attenuates the association between estradiol levels and extinction recall in premenopausal women

**Figure 5:**
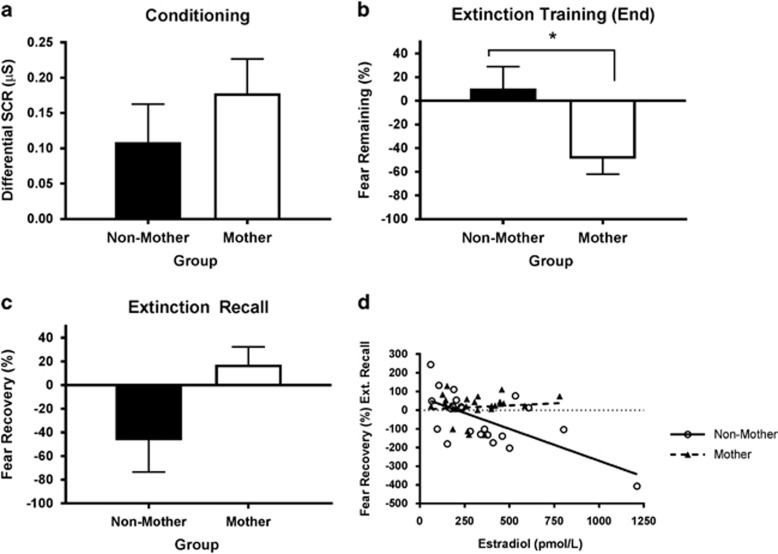
(**a**) Mean (±s.e.m.) differential skin conductance response (SCR; μS) for non-mother (*n*=23) and mother (*n*=23) groups during conditioning in experiment 4. (**b**) Mean (±s.e.m.) fear remaining (%) at the end of extinction training in experiment 4. *Non-mothers > mothers (*P*<0.05). (**c**) Mean (±s.e.m.) fear recovery (%) during extinction recall in experiment 4. (**d**) Mean fear recovery (%) as a function of estradiol (pm/l) during extinction recall in experiment 4.

Finally, we assessed whether our findings could be translated across species by comparing the SCRs of women with and without reproductive experience that underwent extinction training with varying estradiol levels. The groups differed significantly in age (F(1,44)=38.626, *P*<0.001; Supplementary Table S2), but not in estradiol levels (F(1,44)=0.079, *P*=0.780; Supplementary Table S2). The mothers selected a higher shock level than the non-mothers (F(1,44)=11.350, *P*=0.002; Supplementary Table S2). However, the groups did not differ in average UCR (Supplementary Table S2), suggesting that both groups selected a level of shock appropriate to their differing pain thresholds. On Day 1, fear conditioning strength did not differ (F(1,44)=0.901, *P*=0.348; Figure 5a), and was not significantly correlated with estradiol (*r*=0.130, *P*=0.389). However, the mothers exhibited better extinction acquisition than the non-mothers (F(1,44)=5.628, *P*=0.022; Figure 5b), although there was no correlation with estradiol (*r*=0.0.43, *P*=0.776). On Day 2, percent recovery during extinction did not differ (F(1,44)=3.607, *P*=0.064; Figure 5c), although estradiol was significantly negatively correlated with percent recovery (*r*=−0.465, *P*=0.001). Controlling for age, shock level and extinction acquisition, the multiple regression analysis revealed no effect of reproductive status (*P*=0.219), but a significant effect of estradiol on percent recovery (*P*=0.001), with lower levels associated with higher percentage fear recovery. There was also a significant reproductive experience × estradiol interaction (*P*=0.010). Separate correlations between estradiol and percent recovery revealed that, although there was a significant negative correlation for non-mothers (*r*=−0.659, *P*=0.001), there was no correlation for mothers (*r*=0.103, *P*=0.639; Figure 5d).

## Discussion

The present experiments demonstrate that reproductive experience alters the endocrine and behavioral features of fear extinction in female rats and women; effects that persist long after the hormonal surges of pregnancy and lactation have diminished. The metestrus phase of the estrous cycle was associated with impaired extinction acquisition (experiment 1) and/or extinction recall (experiments 1–3) in nulliparous rats. Such variation replicates the existing literature, with reports of metestrus-associated impairments in both extinction acquisition and recall^6, 7^ or extinction recall alone^5, 8^ in nulliparous females. We found that a single reproductive experience mitigated this association between estrous cycle phase and extinction recall (experiment 1), even when fear extinction occurred 3 months after pups had been weaned (experiments 2 and 3). These findings translated to humans—in contrast to non-mothers, there was no association between serum estradiol and extinction recall in mothers, on average, 4 years following the birth of their youngest child (experiment 4). Moreover, although nulliparous rats displayed renewal and reinstatement, these common relapse phenomena were absent in primiparous rats (experiments 2 and 3).

These findings lead us to speculate that reproductive experience may alter the endocrine mechanisms that regulate fear extinction. In nulliparous females, fear extinction is dependent on ER activation,^7^ which may account for the association between natural fluctuations in peripheral estradiol and extinction recall in nulliparous rats and young women (that is, non-mothers).^3, 4^ However, the present experiments demonstrated that reproductively experienced rats and women display comparable extinction recall, whether extinguished during the high or low estradiol phases of the estrous/menstrual cycle. Previous research has shown that primiparous rats have decreased estradiol levels compared with nulliparous rats during proestrus^25^ and human mothers have lower estradiol levels than non-mothers across the menstrual cycle.^26^ Our results from experiment 2 replicated the finding that primiparous rats exhibit reduced serum estradiol levels relative to nulliparous rats during proestrus. Unexpectedly, we found no differences in serum estradiol between metestrus- and proestrus-primiparous rats, despite obtaining the expected differences in serum estradiol between metestrus- and proestrus-nulliparous rats and despite both nulliparous and primiparous rats exhibiting normal cycling according to vaginal cytology. To our knowledge, no previous research has compared serum estradiol levels in primiparous rats during different estrous cycle phases; however, it is possible that as peak estradiol levels are reduced following motherhood,^25, 26^ the further reduction in estradiol during metestrus could be more difficult to detect. In addition, estradiol may reach peak levels during proestrus for a shorter duration following motherhood, narrowing the time frame within which to detect cyclic differences. Irrespective of the exact explanation, our data clearly show that peripheral estradiol levels in primiparous rats are comparable to those of nulliparous rats during the metestrus phase of the estrous cycle. So, although low peripheral estradiol is associated with impaired extinction in nulliparous females, it seems to have no association with extinction in reproductively experienced female rats and women. Nevertheless, there did appear to be increased variation in extinction recall among primiparous rats, which was at times comparable to that of proestrus-nulliparous rats (experiments 2 and 3), and at other times, midway between proestrus- and metestrus-nulliparous rats (experiment 1). This variation did not correspond with estrous stage, and its underlying source is unclear.

It is possible that reproductive experience causes extinction to depend on a lower level of peripheral estradiol than that required by nulliparous females. Indeed, there is evidence to suggest that reproductive experience results in increased neural sensitivity to estradiol. Hippocampal cell proliferation is upregulated by estradiol in multiparous compared with nulliparous female rats,^27^ which may be owing to long-lasting changes in ER expression^28^ and activation^29^ following reproductive experience. Alternatively, reproductive experience may shift the hormonal mechanisms underlying extinction from estrogen-dependent to estrogen-independent. Future studies could assess these hypotheses by comparing the impact of differing doses of ER agonists and antagonists on extinction recall in primiparous and nulliparous rats.

In addition to the attenuation of estrous cycle influences on extinction, we observed an absence of renewal and reinstatement in primiparous rats. Although such findings are surprising, prior studies have revealed similar instances of resistance to fear relapse. For example, juvenile male rodents do not show renewal or reinstatement until post-weaning.^30^ Similarly, an isolated CS presentation 1 h before extinction training prevents renewal and reinstatement in adult male rats ^31^ and humans.^32^ In both cases, the underlying neurobiological substrates of extinction appear to differ from that underlying ‘typical' fear extinction (that which is associated with renewal and reinstatement). For example, in contrast to adult male rats,^13^ extinction in juvenile males does not depend on the infralimbic cortex,^33^ or NMDA^34^ and GABA^35^ receptor activation. Likewise, although extinction in adult male rats leads to phosphorylation of the GluR1 receptor in the lateral amygdala, extinction that follows an isolated CS presentation leads to GluR1 dephosphorylation.^31^ Therefore, it is conceivable that major life events, such pregnancy and motherhood, could result in similar alterations in the neurobiology of extinction, which in turn may promote relapse resistance. This remains to be tested in future studies.

Another question of interest is whether the effects of reproductive experience on fear extinction are due to pregnancy, maternal experience or a combination of both. Previous research on this issue has produced equivocal answers. For example, primiparous rats whose pups were removed at birth were no longer protected against the detrimental effects of stress on eyeblink conditioning; conversely, such protection was induced in nulliparous rats exposed to foster pups.^23^ Likewise, reproductive experience-induced improvements in spatial learning were attenuated by pup removal, and induced in nulliparous rats by pup fostering.^36, 37^ These findings suggest that maternal experience, rather than pregnancy, may be important for the changes in stress resilience and spatial learning following motherhood. Another study, however, reported that the enhanced spatial learning in nulliparous rats exposed to foster pups was no longer evident 1 month after the period of pup exposure.^37^ That is, the effects of maternal experience alone may only be temporary, whereas the changes associated with both pregnancy and maternal experience are longer-lasting.

Identifying whether the changes in fear extinction observed in the present experiments are due to pregnancy or maternal experience (or both) may help to identify candidate endocrine mechanisms underlying such changes. For example, peripheral levels of estradiol and progesterone are persistently elevated throughout the 23-day gestation period. Transient fluctuations (~24 h) in these hormones across the estrous cycle correspond with pronounced neural changes, such as increased hippocampus dendritic spine density during proestrus, which revert when hormonal levels decline.^38^ The substantially increased exposure to estradiol and progesterone during pregnancy may have more pronounced and longer-term consequences, like the effects on fear extinction in primiparous rats. In addition, circulating levels of prolactin and oxytocin increase during the postpartum period, and are crucial for lactation as well as maternal behaviors responsible for mother–infant bonding. Downregulation of prolactin receptor expression blocked the attenuation of stress reactivity in primiparous female rats during the postpartum period,^39^ and intracerebral infusions of prolactin induced attenuated stress reactivity in nulliparous rats.^40^ Likewise, an oxytocin antagonist prevented the enhanced spatial learning normally observed in multiparous mice, and intracerebral infusions of oxytocin improved spatial learning in nulliparous mice.^41^ Together, these studies suggest that postpartum surges in prolactin and oxytocin mediate changes in threat responsivity and learning/memory induced by reproductive experience. Similarly, these hormones may partly underlie the endocrine and behavioral changes in fear extinction associated with reproductive experience observed in the present experiments.

Anxiety disorders are twice more common in women than men, and are associated with a greater burden on women's health and well-being.^42^ It is therefore alarming that fear extinction in females has become a focus of research only in recent years.^4^ The present experiments add to this growing body of literature by aiding the development of a more ecologically valid cross-species model of extinction that accounts for reproductive experience, in addition to hormonal status, in females. Our cross-species findings suggest that the hormonal and behavioral characteristics of fear extinction may change significantly following motherhood. Further studies are needed to assess which of the hormonal and environmental factors associated with motherhood could help to protect against dysregulated fear inhibition in women.
