# Supplementary material for: Mothers do it differently: reproductive experience alters fear extinction in female rats and women
Source: Transl Psychiatry. 2016 Oct 25;6(10):e928–. doi: 10.1038/tp.2016.193 (PMC5290346; doi:10.1038/tp.2016.193)
Supplement: Supplementary Information [file tp2016193x1.docx]

**Mothers do it differently: reproductive experience alters fear extinction in female rats and women**

***Supplemental Information***

**Supplemental Methods and Materials**

*Animal Subjects*

Rats were maintained on a 12-hour light/dark cycle, and food and water were available *ad libitum*. For 3 consecutive days, rats were handled for 5 minutes per day, and pre-exposed to the conditioning apparatus for 15 minutes per day.

*Breeding*

For 2.5 weeks, groups of 4 to 5 rats designated as breeders (primiparous group) were housed in “harem” boxes (67 cm long × 30 cm wide × 22 cm high) with a single sexually experienced male rat. Once removed from the “harem”, pregnant rats were individually housed in “litter” boxes (24.5cm long x 37cm wide x 27 cm high). Approximately 22 days after insemination (1), rats gave birth to litters ranging in size from 10 to 16 pups. At postnatal day (PND) 1, litters were culled to 8 pups, with the majority of the spared pups being males. To enable a natural mothering experience, primiparous rats were housed with their litters until pups were weaned at PND 24.

*Vaginal Smears*

Vaginal smears were used to determine estrous cycle phase. Rats have a 4-day estrous cycle that contains 4 phases named estrus, metestrus, diestrus and proestrus. Each of these phases has a distinct hormonal profile and vaginal cytology, and so estradiol levels can be inferred by examining vaginal epithelial cells (2).

Vaginal smears were performed daily between 8:00am and 10:00am, starting at least 4 days before fear conditioning and ending 3 days after test. A cotton-tip moistened with 0.9% saline solution was inserted into the vaginal canal and then rapidly twisted to collect epithelial cells. These cells were transferred to a microscope slide and dyed with a KwikDiff Stain Kit (Thermo Fisher Scientific, Australia). The slide was then inspected under a light microscope at 10× magnification. Estrous cycle phase was identified by the presence of nucleated cells (proestrus), cornified cells (estrus), and leukocytes (diestrus), as well as the absence of leukocytes (metestrus). A blind observer cross-checked a random sample of slides to ensure proper identification of estrous cycle phase.

*Apparatus*

Two sets of two identical experimental chambers (24 cm long × 30 cm wide × 21 cm high) were used for conditioning, extinction and test procedures. All four chambers were housed in separate wooden cabinets to minimise external auditory and visual stimulation; however, ventilation fans provided low, constant background noise. An infrared video camera mounted on the rear wall of the cabinets recorded the behaviour of each rat inside the chamber. The chambers were regularly wiped clean with tap water.

The two sets of experimental chambers differed in a number of visual and tactile features, and so served as distinct contexts for experimental procedures. The first set of two chambers was designated Context A, and all rats were conditioned in this context. The front walls, rear walls and ceilings of these chambers were constructed of clear Perspex. The sidewalls were made of stainless steel, and one was embedded with a high-frequency speaker. The floor consisted of stainless steel rods set 1.5 cm apart, and these were connected to a shock generator. These chambers were illuminated by infrared light from the video camera.

The second set of two chambers was designated Context B, and all rats were extinguished and tested in this context. These chambers differed from Context A in that only the rear walls were constructed of clear Perspex. The front walls were covered with a piece of patterned paper (2.5 cm wide vertical black and white stripes), and the ceilings were overlaid with a sheet of opaque Perspex. The stainless steel rod floor was also covered with a sheet of opaque Perspex. White light from a table lamp illuminated these chambers.

*Behavioral Data Analysis*

Rats were scored as freezing or not freezing every 3 seconds during the adaptation periods and CS presentations of fear conditioning, extinction training and extinction recall. A percentage score was calculated to determine the proportion of total observations spent freezing. A random sample of data was cross-scored by a second observer who was blind to the experimental condition of the rats. The inter-rater reliability exceeded .9.

*Serological Estradiol Measurements*

Naturally-cycling women were invited to participate in the experiment across all phases of the menstrual cycle to achieve wide variance in estradiol levels. A blood sample was drawn from each participant by a registered nurse approximately 15 minutes after extinction training. Their estradiol levels were assessed by a partner laboratory of Healthscope Pathology, and results were available within 24 hours. Estradiol levels were analysed using an ADVIA Centaur Enhanced Estradiol competitive immunoassay (Siemens, Australia), with a measuring range of 43.6-11 010 pmol/L.

*Conditioned and Unconditioned Stimuli*

The conditioned stimuli (CSs) were two black and white photographs of male faces with neutral expressions taken from the Center for Vital Longevity Face database (3). The CSs were presented on a Dell PC using E-Prime software in a pseudo-random order. On each CS trial, a grey rectangle was presented for 2 s and then the CS was presented for 6 s. The inter-trial interval was 10 s.

The unconditioned stimulus was a 0.5 s mild electric shock, the level of which was selected by participants to be “highly annoying but not painful” (minimum 3.9mA; maximum 30mA). The US was controlled by a second Dell PC using E-Prime software that was connected to a customised ADInstruments constant current stimulus isolator running on Labchart software. The shock was delivered through a set of stainless steel dry bipolar electrodes (MLT116F) that were Velcro-strapped to the distal phalanx of the index and middle fingers of the dominant hand.

*Psychophysiology Amp*

Skin conductance levels (SCLs) were recorded by an ADInstruments galvanic skin response amp (FE116) using constant voltage (22 mV_rms_ at 75 Hz) AC excitation through another set of stainless steel dry bipolar electrodes (MLT116F) that were Velcro-strapped to the same fingertips of the non-dominant hand. The analogue inputs were digitized by an ADInstruments Powerlab 8/35 data acquisition system (PL3508), and sampled using Labchart.

*Rating Scales*

Rating scales were used to examine participants’ fear of the faces and expectancy of the shock during each of the four experimental phases. These data are not presented, as the results are subsidiary to the central research question, but are available upon request from the corresponding author.

*Psychophysiological Data Analysis*

Skin conductance responses (SCRs) were used as the measure of conditioned fear. SCRs were square-root transformed to reduce heteroscedasticity. Conditioned SCRs were calculated by subtracting the average SCL during the 2 s presentation of the grey rectangle that preceded the CS from the maximum SCR during the 6 s presentation of the CS. This was to ensure that SCR values represented changes over and above those produced by visual stimulation alone, and that the maximum increase in SCL during the 6 s presentation of the CS would be detected (4). Unconditioned SCRs were calculated by subtracting the average SCL during the first 1.5 s after the shock from the maximum SCL during the 5 s after the shock.

Conditioning strength was assessed by calculating the average differential SCRs across conditioning trials. To do this, the average SCRs to the CS- was subtracted from the average SCRs to the CS+. Extinction acquisition was assessed by calculating the percentage of fear remaining after extinction as a function of conditioning acquisition. This was achieved by dividing the average SCRs to the CS+ during the last two extinction trials by the maximum SCR to the CS+ during conditioning, and multiplying the result by 100. Extinction recall was assessed by calculating the percentage of fear recovered during test as a function of conditioning acquisition. This was achieved by dividing the average SCRs to the CS+ during the first two test trials by the maximum SCR to the CS+ during conditioning, and multiplying the result by 100.

*Statistical Analysis*

Three statistical outliers were removed from the analysis of experiment 1: one nulliparous-proestrus rat because baseline freezing prior to extinction training was 9.55 SDs above the mean; one nulliparous-metestrus rat because CS-elicited freezing during the first block of extinction training was 4.02 SDs below the mean; and one nulliparous-proestrus rat because CS-elicited freezing during the final block of extinction training was 4.37 SDs above the mean. One nulliparous-metestrus rat was removed from the serum analyses of experiment 3 serum estradiol was 4 SDs below the mean.

Three statistical outliers were removed from the analysis of experiment 4: one non-mother because within-session extinction was 12.02 SDs above the mean; one mother because extinction recall was 19.04 SDs above the mean; and another non-mother whose serum estradiol levels were 4.01 SDs above the mean.

**Supplemental Results**

*Experiment 1*

Prior to fear conditioning, no rats exhibited freezing. Prior to extinction training, there were no significant effects on baseline freezing [largest *F*(1, 38)=.842, *p*=.365]. Prior to extinction recall, there was a significant reproductive experience × estrous cycle interaction on baseline freezing [*F*(1,38)=5.958, *p*=.019], due to nulliparous-proestrus rats exhibiting no (0%) baseline freezing.

*Experiment 2*

Prior to fear conditioning, no rats exhibited freezing. Prior to extinction training, there were no main effects of reproductive experience or estrous cycle, and no reproductive experience × estrous cycle interaction for baseline freezing [largest *F*(1,35)=2.51, *p*=.122]. Prior to extinction recall in the extinction context, there were no main effects of groups or interaction between groups for baseline freezing [largest *F*(1,35)=1.78, *p*=.194]. Prior to the renewal test in the conditioning context, there were no main effects of groups or interaction between groups for baseline freezing [largest *F*(1,35)=1.22, *p*=.278].

*Experiment 3*

Prior to fear conditioning, no rats exhibited freezing. Prior to extinction training, there were no main effects of reproductive experience or estrous cycle, but there was a significant reproductive experience × estrous cycle interaction on baseline freezing [*F*(1,31)=4.43, *p*=.044]. Follow-up tests revealed no group differences underlying this interaction. Prior to extinction recall, there were no main effects of groups or interaction between groups for baseline freezing [largest *F*(1,31)=.03, *p*=.87]. During the baseline period prior to the reinstating shock, there were no main effects of groups or interactions between the groups for freezing levels [largest *F*(1,31)=1.14, *p*=.29]. Prior to reinstatement test, there were no main effects of groups or interaction between groups for baseline freezing [largest *F*(1,31)=2.74, *p*=.11].

**Table S1.** Baseline freezing during the adaptation period in Experiments 1, 2 and 3.

**NP-Pro NP-Met PP-Pro PP-Met**

**M (SEM) M (SEM) M (SEM) M (SEM)**

Experiment 1 *n* = 10 *n* = 10 *n* = 12 *n* = 10

Conditioning 0 (0) 0 (0) 0 (0) 0 (0)

Extinction 4.3 (2.4) 5.5 (2.5) 4.4 (1.6) 2.0 (1.0)

Recall Test 0.0 (0.0) 5.0 (2.8) 8.3 (2.5) 3.0 (1.7)

Experiment 2 *n* = 11 *n* = 11 *n* = 9 *n* = 8

Conditioning 0 (0) 0 (0) 0 (0) 0 (0)

Extinction 4.0 (1.9) 5.4 (2.6) 2.2 (1.7) 0.8 (0.5)

Recall Test 0.9 (0.6) 3.6 (1.9) 1.1 (1.1) 1.9 (0.9)

Renewal Test 3.2 (2.3) 1.7 (0.7) 3.3 (2.8) 0.6 (0.6)

Experiment 3 *n* = 8 *n* = 8 *n* = 10 *n* = 9

Conditioning 0 (0) 0 (0) 0 (0) 0 (0)

Extinction 0.4 (0.4) 16.8 (9.7) 8.6 (4.9) 2.6 (1.5)

Recall Test 0.6 (0.6) 0.6 (0.6) 0.5 (1.6) 0.6 (0.6)

Reinstating Shock 1.4 (1.0) 5.8 (4.2) 2.5 (1.7) 3.2 (1.7)

Reinstatement Test 4.4 (2.2) 18.1 (4.9) 10.5 (8.4) 5.6 (2.8)

NP = nulliparous; PP = primiparous; Pro = proestrus; Met = metestrus.

**Table S2.** Demographic and baseline psychophysiological results for Experiment 4

**Non-Mother Mother**

***n* = 23 *n* = 23**

**M (SEM) M (SEM)**

Age **24.0 (1.1) 34.0 (1.2)**

No. of Children N.A. 1.6 (0.2)

Age of Children N.A. 3.9 (0.9)

Estradiol Level 337.4 (55.8) 319.0 (34.5)

Shock Level **6.2 (0.6) 11.4 (1.4)**

Baseline SCL 4.6 (0.1) 4.7 (0.1)

Average UCR 1.1 (0.1) 1.2 (0.1)

No. = number; SCL = skin conductance level; UCR = unconditioned response. Bolded values indicate *p* < 0.05.

**Supplemental References**

1. Love G, Torrey N, McNamara I, Morgan M, Banks M, Hester NW, et al. (2005): Maternal experience produces long-lasting behavioral modifications in the rat. *Behav Neurosci*. 119:1084-1096.

2. Becker JB, Arnold AP, Berkley KJ, Blaustein JD, Eckel LA, Hampson E, et al. (2005): Strategies and methods for research on sex differences in brain and behavior. *Endocrinology*. 146:1650-1673.

3. Minear M, Park DC (2004): A lifespan database of adult facial stimuli. *Behav Res Methods Instrum Comput*. 36:630-633.

4. Milad MR, Orr SP, Pitman RK, Rauch SL (2005): Context modulation of memory for fear extinction in humans. *Psychophysiology*. 42:456-464.
